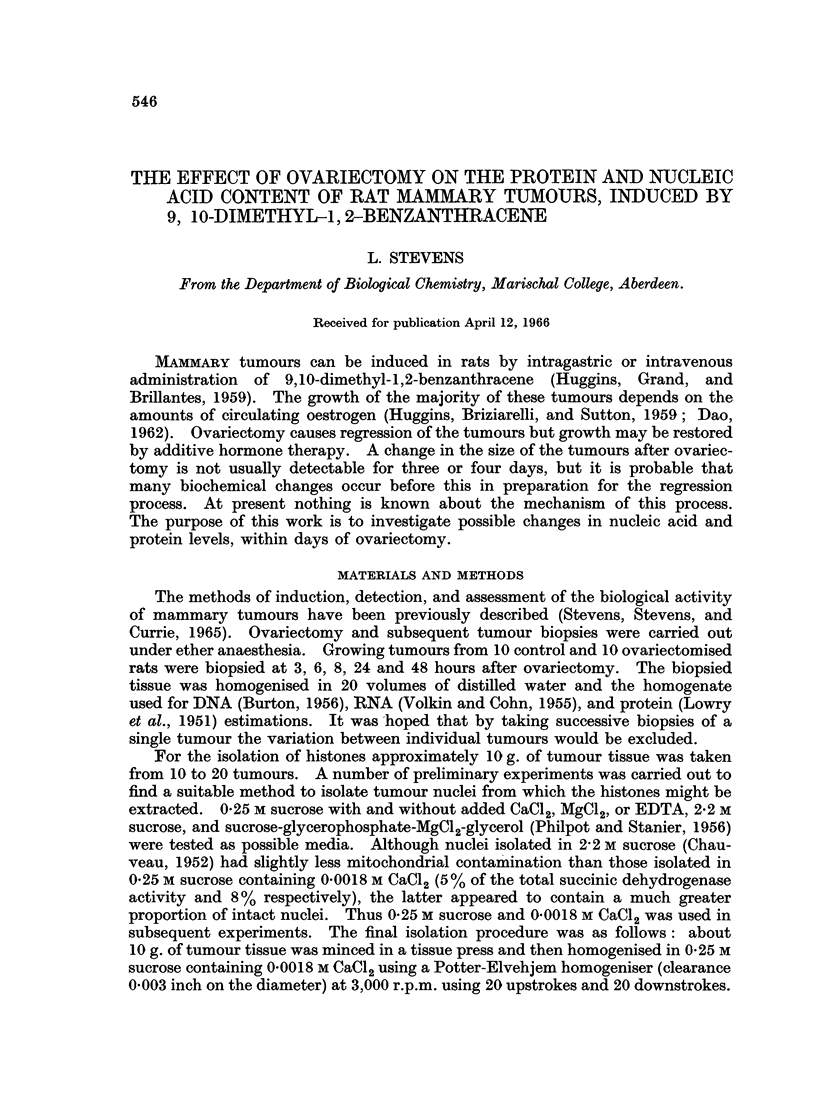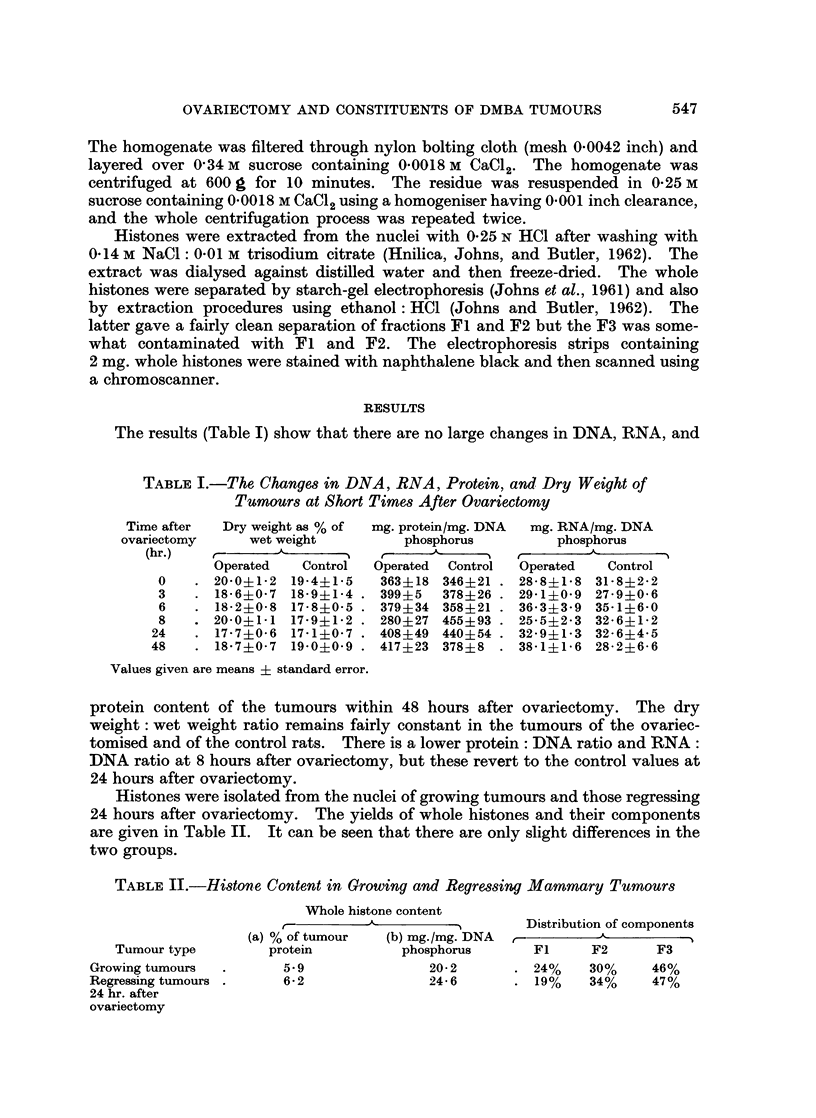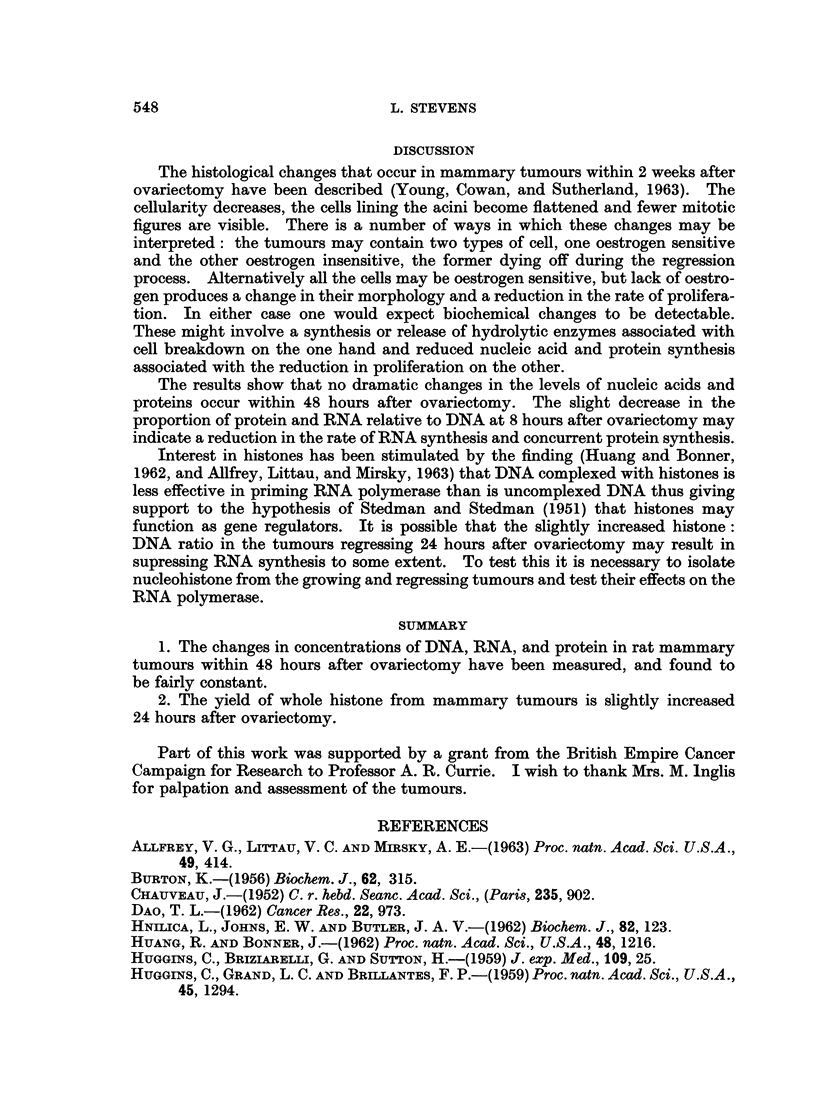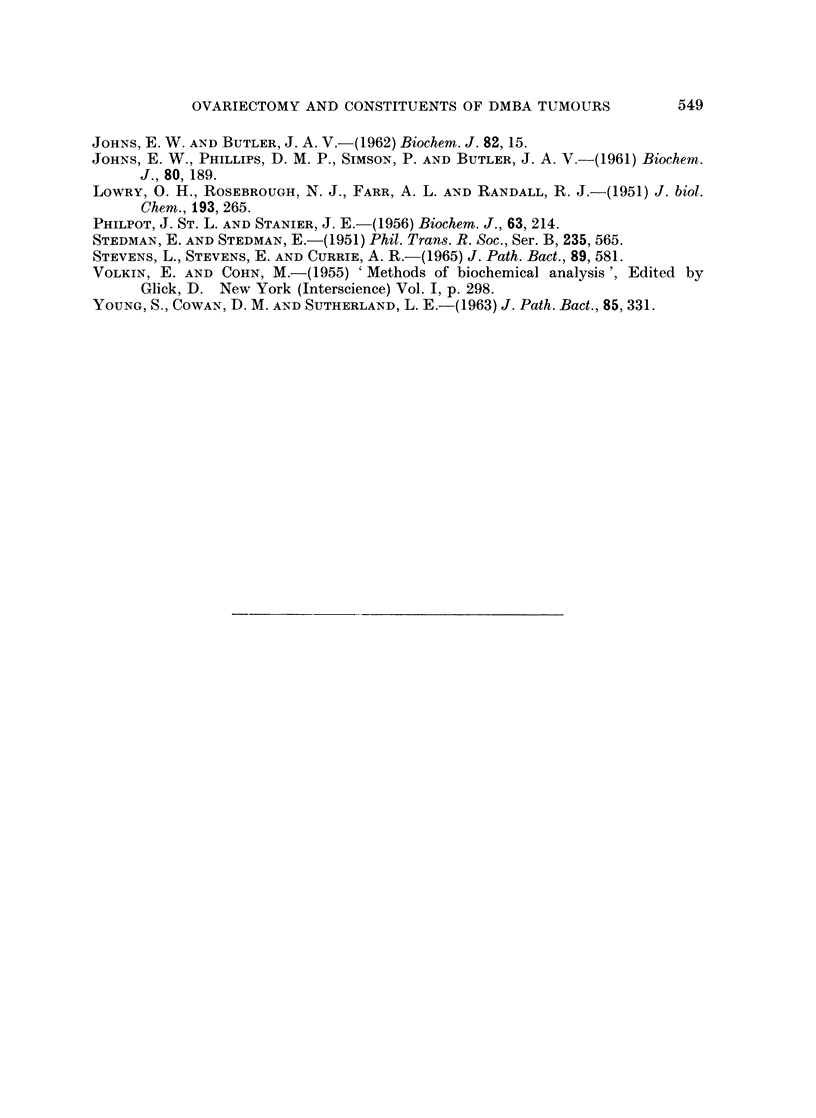# The effect of ovariectomy on the protein and nucleic acid content of rat mammary tumours, induced by 9, 10-dimethyl-1,2-benzanthracene.

**DOI:** 10.1038/bjc.1966.67

**Published:** 1966-09

**Authors:** L. Stevens


### THE EFFECT OF OVARIECTOMY ON THE PROTEIN AND NUCLEIC ACID CONTENT OF RAT MAMMARY TUMOURS, INDUCED BY 9, 10-DIMETHYL-1, 2-BENZANTHRACENE

L. STEVENS

*From the Department of Biological Chemistry, Marischal College, Aberdeen.*

Received for publication April 12, 1966

MAMMARY tumours can be induced in rats by intragastric or intravenous administration of 9,10-dimethyl-1,2-benzanthracene (Huggins, Grand, and Brillantes, 1959). The growth of the majority of these tumours depends on the amounts of circulating oestrogen (Huggins, Briziarelli, and Sutton, 1959; Dao, 1962). Ovariectomy causes regression of the tumours but growth may be restored by additive hormone therapy. A change in the size of the tumours after ovariectomy is not usually detectable for three or four days, but it is probable that many biochemical changes occur before this in preparation for the regression process. At present nothing is known about the mechanism of this process. The purpose of this work is to investigate possible changes in nucleic acid and protein levels, within days of ovariectomy.

### MATERIALS AND METHODS

The methods of induction, detection, and assessment of the biological activity of mammary tumours have been previously described (Stevens, Stevens, and Currie, 1965). Ovariectomy and subsequent tumour biopsies were carried out under ether anaesthesia. Growing tumours from 10 control and 10 ovariectomised rats were biopsied at 3, 6, 8, 24 and 48 hours after ovariectomy. The biopsied tissue was homogenised in 20 volumes of distilled water and the homogenate used for DNA (Burton, 1956), RNA (Volkin and Cohn, 1955), and protein (Lowry *et al.*, 1951) estimations. It was hoped that by taking successive biopsies of a single tumour the variation between individual tumours would be excluded.

For the isolation of histones approximately 10 g. of tumour tissue was taken from 10 to 20 tumours. A number of preliminary experiments was carried out to find a suitable method to isolate tumour nuclei from which the histones might be extracted. 0·25 M sucrose with and without added $CaCl_2$, $MgCl_2$, or EDTA, 2·2 M sucrose, and sucrose-glycerophosphate-$MgCl_2$-glycerol (Philpot and Stanier, 1956) were tested as possible media. Although nuclei isolated in 2·2 M sucrose (Chauveau, 1952) had slightly less mitochondrial contamination than those isolated in 0·25 M sucrose containing 0·0018 M $CaCl_2$ (5% of the total succinic dehydrogenase activity and 8% respectively), the latter appeared to contain a much greater proportion of intact nuclei. Thus 0·25 M sucrose and 0·0018 M $CaCl_2$ was used in subsequent experiments. The final isolation procedure was as follows: about 10 g. of tumour tissue was minced in a tissue press and then homogenised in 0·25 M sucrose containing 0·0018 M $CaCl_2$ using a Potter-Elvehjem homogeniser (clearance 0·003 inch on the diameter) at 3,000 r.p.m. using 20 upstrokes and 20 downstrokes.

The homogenate was filtered through nylon bolting cloth (mesh 0·0042 inch) and layered over 0·34 M sucrose containing 0·0018 M $CaCl_2$. The homogenate was centrifuged at 600 g for 10 minutes. The residue was resuspended in 0·25 M sucrose containing 0·0018 M $CaCl_2$ using a homogeniser having 0·001 inch clearance, and the whole centrifugation process was repeated twice.

Histones were extracted from the nuclei with 0·25 N HCl after washing with 0·14 M NaCl : 0·01 M trisodium citrate (Hnilica, Johns, and Butler, 1962). The extract was dialysed against distilled water and then freeze-dried. The whole histones were separated by starch-gel electrophoresis (Johns *et al.*, 1961) and also by extraction procedures using ethanol : HCl (Johns and Butler, 1962). The latter gave a fairly clean separation of fractions F1 and F2 but the F3 was somewhat contaminated with F1 and F2. The electrophoresis strips containing 2 mg. whole histones were stained with naphthalene black and then scanned using a chromoscanner.

### RESULTS

The results (Table I) show that there are no large changes in DNA, RNA, and protein content of the tumours within 48 hours after ovariectomy. The dry weight : wet weight ratio remains fairly constant in the tumours of the ovariectomised and of the control rats. There is a lower protein : DNA ratio and RNA : DNA ratio at 8 hours after ovariectomy, but these revert to the control values at 24 hours after ovariectomy.

TABLE I.—*The Changes in DNA, RNA, Protein, and Dry Weight of Tumours at Short Times After Ovariectomy*

| Time after ovariectomy (hr.) | Dry weight as % of wet weight | | mg. protein/mg. DNA phosphorus | | mg. RNA/mg. DNA phosphorus | |
|---|---|---|---|---|---|---|
| | Operated | Control | Operated | Control | Operated | Control |
| 0 | 20·0±1·2 | 19·4±1·5 | 363±18 | 346±21 | 28·8±1·8 | 31·8±2·2 |
| 3 | 18·6±0·7 | 18·9±1·4 | 399±5 | 378±26 | 29·1±0·9 | 27·9±0·6 |
| 6 | 18·2±0·8 | 17·8±0·5 | 379±34 | 358±21 | 36·3±3·9 | 35·1±6·0 |
| 8 | 20·0±1·1 | 17·9±1·2 | 280±27 | 455±93 | 25·5±2·3 | 32·6±1·2 |
| 24 | 17·7±0·6 | 17·1±0·7 | 408±49 | 440±54 | 32·9±1·3 | 32·6±4·5 |
| 48 | 18·7±0·7 | 19·0±0·9 | 417±23 | 378±8 | 38·1±1·6 | 28·2±6·6 |

Values given are means ± standard error.

Histones were isolated from the nuclei of growing tumours and those regressing 24 hours after ovariectomy. The yields of whole histones and their components are given in Table II. It can be seen that there are only slight differences in the two groups.

TABLE II.—*Histone Content in Growing and Regressing Mammary Tumours*

| Tumour type | Whole histone content: (a) % of tumour protein | Whole histone content: (b) mg./mg. DNA phosphorus | Distribution of components: F1 | F2 | F3 |
|---|---|---|---|---|---|
| Growing tumours | 5·9 | 20·2 | 24% | 30% | 46% |
| Regressing tumours 24 hr. after ovariectomy | 6·2 | 24·6 | 19% | 34% | 47% |

### DISCUSSION

The histological changes that occur in mammary tumours within 2 weeks after ovariectomy have been described (Young, Cowan, and Sutherland, 1963). The cellularity decreases, the cells lining the acini become flattened and fewer mitotic figures are visible. There is a number of ways in which these changes may be interpreted : the tumours may contain two types of cell, one oestrogen sensitive and the other oestrogen insensitive, the former dying off during the regression process. Alternatively all the cells may be oestrogen sensitive, but lack of oestrogen produces a change in their morphology and a reduction in the rate of proliferation. In either case one would expect biochemical changes to be detectable. These might involve a synthesis or release of hydrolytic enzymes associated with cell breakdown on the one hand and reduced nucleic acid and protein synthesis associated with the reduction in proliferation on the other.

The results show that no dramatic changes in the levels of nucleic acids and proteins occur within 48 hours after ovariectomy. The slight decrease in the proportion of protein and RNA relative to DNA at 8 hours after ovariectomy may indicate a reduction in the rate of RNA synthesis and concurrent protein synthesis.

Interest in histones has been stimulated by the finding (Huang and Bonner, 1962, and Allfrey, Littau, and Mirsky, 1963) that DNA complexed with histones is less effective in priming RNA polymerase than is uncomplexed DNA thus giving support to the hypothesis of Stedman and Stedman (1951) that histones may function as gene regulators. It is possible that the slightly increased histone : DNA ratio in the tumours regressing 24 hours after ovariectomy may result in supressing RNA synthesis to some extent. To test this it is necessary to isolate nucleohistone from the growing and regressing tumours and test their effects on the RNA polymerase.

### SUMMARY

1. The changes in concentrations of DNA, RNA, and protein in rat mammary tumours within 48 hours after ovariectomy have been measured, and found to be fairly constant.

2. The yield of whole histone from mammary tumours is slightly increased 24 hours after ovariectomy.

Part of this work was supported by a grant from the British Empire Cancer Campaign for Research to Professor A. R. Currie. I wish to thank Mrs. M. Inglis for palpation and assessment of the tumours.

### REFERENCES

ALLFREY, V. G., LITTAU, V. C. AND MIRSKY, A. E.—(1963) *Proc. natn. Acad. Sci. U.S.A.*, **49**, 414.

BURTON, K.—(1956) *Biochem. J.*, **62**, 315.

CHAUVEAU, J.—(1952) *C. r. hebd. Seanc. Acad. Sci.*, (*Paris*, **235**, 902.

DAO, T. L.—(1962) *Cancer Res.*, **22**, 973.

HNILICA, L., JOHNS, E. W. AND BUTLER, J. A. V.—(1962) *Biochem. J.*, **82**, 123.

HUANG, R. AND BONNER, J.—(1962) *Proc. natn. Acad. Sci., U.S.A.*, **48**, 1216.

HUGGINS, C., BRIZIARELLI, G. AND SUTTON, H.—(1959) *J. exp. Med.*, **109**, 25.

HUGGINS, C., GRAND, L. C. AND BRILLANTES, F. P.—(1959) *Proc. natn. Acad. Sci., U.S.A.*, **45**, 1294.

JOHNS, E. W. AND BUTLER, J. A. V.—(1962) *Biochem. J.* **82**, 15.
JOHNS, E. W., PHILLIPS, D. M. P., SIMSON, P. AND BUTLER, J. A. V.—(1961) *Biochem. J.*, **80**, 189.
LOWRY, O. H., ROSEBROUGH, N. J., FARR, A. L. AND RANDALL, R. J.—(1951) *J. biol. Chem.*, **193**, 265.
PHILPOT, J. ST. L. AND STANIER, J. E.—(1956) *Biochem. J.*, **63**, 214.
STEDMAN, E. AND STEDMAN, E.—(1951) *Phil. Trans. R. Soc.*, Ser. B, **235**, 565.
STEVENS, L., STEVENS, E. AND CURRIE, A. R.—(1965) *J. Path. Bact.*, **89**, 581.
VOLKIN, E. AND COHN, M.—(1955) 'Methods of biochemical analysis', Edited by Glick, D. New York (Interscience) Vol. I, p. 298.
YOUNG, S., COWAN, D. M. AND SUTHERLAND, L. E.—(1963) *J. Path. Bact.*, **85**, 331.